# Injections and infections: understanding syringe service program utilization in a rural state

**DOI:** 10.1186/s12954-021-00524-1

**Published:** 2021-07-17

**Authors:** Kinna Thakarar, Nitysari Sankar, Kimberly Murray, Frances L. Lucas, Debra Burris, Robert P. Smith

**Affiliations:** 1 Center for Outcomes Research and Evaluation/Maine Medical Center Research Institute, 509 Forest Ave, Portland, ME USA; 2 Department of Medicine, Maine Medical Center, 22 Bramhall Street, Portland, ME USA; 3 Tufts University School of Medicine, 145 Harrison Ave, Boston, MA USA; 4 University of New England College of Osteopathic Medicine, Biddeford, ME USA; 5 Maine Medical Partners Adult Infectious Diseases, 41 Donald Bean Drive, Suite B, South Portland, ME 04106 USA

**Keywords:** Injections and infections, Syringe Service, Rural State, Injection drug use (IDU), Syringe service program (SSP)

## Abstract

**Background:**

Increasing rates of injection drug use (IDU) associated-infections suggest significant syringe service program (SSP) underutilization. Our study objective was to assess practices of safe injection techniques and to determine predictors of SSP utilization in a rural state.

**Patients and methods:**

This was a fifteen-month cross-sectional study of participants hospitalized with IDU-associated infections in Maine. Data were collected through Audio Computer-Assisted Self-Interview survey and medical record review. Descriptive analyses were performed to characterize demographics, health characteristics, and injection practices. The primary outcome was SSP utilization, and the main independent variable was self-reported distance to SSP. Logistic regression analyses were performed to identify factors associated SSP utilization, controlling for gender, homelessness, history of overdose, having a primary care physician and distance to SSP.

**Results:**

Of the 101 study participants, 65 participants (64%) reported past 3 month SSP utilization, though only 33% used SSPs frequently. Many participants (57%) lived more than 10 miles from an SSP. Participants who lived less than 10 miles of an SSP were more likely to use an SSP (adjusted odds ratio 5.4; 95% CI 1.9–15.7).

**Conclusions:**

Our study highlights unsafe injection practices and lack of frequent SSP utilization among people admitted with IDU-associated infections in a rural state. Especially given increasing stimulant use, these results also highlight the need for SSP access. Particularly in rural areas where patients may live more than 10 miles from an SSP, expansion of harm reduction services, including mobile units, should be a priority.

## Introduction

The increasing prevalence of substance use disorders in the United States has highlighted the need to examine approaches to reduce injection drug use (IDU)-associated infections [24]. Unsafe injection practices among people who inject drugs (PWID) have led to rising rates of HIV, viral hepatitis, and serious bacterial and fungal infections, such as infective endocarditis [10, 22, 31, 32]. From 2014 to 2018, new HIV diagnoses among PWID in the United States increased by 9% [21] and acute hepatitis C also increased, particularly among people ages 20–39 years old. The number of estimated acute hepatitis C cases in 2018 was 50,300, compared to 17,100 in 2011, with 72% of reported cases attributable to IDU [19]. In a recent study using national commercial and Medicaid databases, the incidence of infective endocarditis among people with opioid use disorder increased from 156.4/100,000 to 642.9/100,000 in 2017 [31]. Harm reduction services, specifically syringe services programs (SSPs), have been shown to effectively counsel clients about safe injection techniques, reduce the transmission of infections like HIV, deliver overdose prevention/education, administer vaccinations, and also facilitate referrals for medication for opioid use disorder (MOUD) [7, 8, 16].

Nationally, outbreaks of HIV and viral hepatitis, such as those in Scott County, Indiana, and the rise of other serious complications such as infective endocarditis, have raised concern about access to preventive and treatment services for PWID in rural areas [5, 14, 15, 33]. Additionally, some factors such as drug availability, close social networks, and economic hardship may play role in the increasing incidence of IDU-associated infections in rural areas [12]. In Maine, a predominantly rural state, the incidence of viral hepatitis and IDU-associated infective endocarditis have increased over recent years. Maine currently has the second highest rate of acute hepatitis B in the United States, with an 11.5% increase in cases from 2018 to 2019. Acute hepatitis C cases in Maine increased by 51.8% from 2018 to 2019, while IDU-associated infective endocarditis cases increased by 25% from 2013 to 2016 [4, 20, 29]. These trends have led to concerns that PWID underutilize SSP services and that SSPs may be less accessible, particularly to rural residents. A recent study showed that a majority of young people with acute hepatitis C in Maine lived ≥ 10 miles from an SSP [5]. The fact that IDU-associated infections have been reported at hospitals both near and far from SSPs suggests that geographic dispersion however may not be the only factor influencing utilization and that other barriers need to be identified. Thus, the goal of our study was to provide a more detailed understanding of factors that influence SSP utilization in Maine and also help inform future interventions to improve access to SSP services, particularly in rural states. Our hypothesis was that distance from SSP would be a significant predictor of SSP utilization, and that other social determinants of health, such as homelessness, insurance and employment status, would play a role as well. Our study objectives were to (1) examine practices around safe injection techniques and (2) determine factors predicting utilization of SSPs.

## Methods

### Study Design

This is a cross-sectional study of participants hospitalized with IDU-associated infections at four hospitals in Maine in counties deemed high risk for HIV/hepatitis outbreaks [30]. A convenience sample of n = 101 participants was prospectively recruited from January 1, 2019 through March 18, 2020. Study enrollment was halted due the COVID-19 pandemic.

### Inclusion and exclusion criteria

Criteria for study enrollment included: (1) inpatient infectious disease consultation for a diagnosis of an IDU-associated infection such as infective endocarditis, skin/soft tissue infection, osteomyelitis, HIV or viral hepatitis, or whose chart has been reviewed by the ID antibiotic stewardship team and found to have an IDU-associated infection; (2) age 18–65; (3) EHR-reported or self-reported injection drug use and/or recent stigmata (e.g., injection sites on physical exam); (4) English speaking; and (5) ability to provide informed consent. Exclusion criteria included intubation, suicidal/homicidal ideation, or if the individual showed signs of psychotic symptoms. Data were collected through Audio Computer-Assisted Self-Interview (ACASI) [1] survey and medical record review. Of 120 individuals eligible for the study based on the inclusion criteria, n = 19 individuals declined to participate. No individuals met exclusion criteria. Upon completion of the survey, which took approximately 30–60 min, study participants were compensated with a $25 gift card for their time and expertise.

### Measurements

#### Outcome

The primary outcome was SSP utilization which was defined as (1) having reported using an SSP in the past 3 months or (2) responding to the question about most common ways the participant accessed an SSP in the past 3 months.

### Variables

#### Independent variable

The main independent variable was distance to closest SSP. Distance from the closest SSP to the participant's address was calculated in miles with an online map tool. If the participant was experiencing homelessness or if the participant's address was not available in electronic health record (EHR), distance in mileage was calculated between the closest SSP and the self-reported place/zip code where the participant most frequently lived or slept in the past 90 days.

#### Covariates

Some variables were collected through self-report. Self-report demographic and health variables included gender, history of incarceration, willingness to take pre-exposure prophylaxis for HIV/discussed pre-exposure prophylaxis with provider, condomless sex, and homelessness. Self-report variables regarding substance use included overdose history and injectable and non-injectable drug(s) of choice. Severity of opioid use disorder was measured using the Short-inventory of Problems-Modified for Drug Use (SIP-DU) [3]**.** *The Bacterial Skin Index Risk Score (BIRSI) score*, which includes questions about alcohol pad and sterile water use, handwashing, rotating injection sites, injecting subcutaneously or in the muscle (“skin/muscle popping”), and clean needle use, was used as a continuous score to measure risk of skin and soft tissue infections [17]. Syringe acquisition and disposal variables (i.e. peer exchange, disposal of needle/syringes in SSPs, public places, etc.) were also collected through self-report. *Unhealthy alcohol use* was categorized using the AUDIT-C score [25]. *Naloxone uptake* was defined by self-report use of naloxone on another person, or having naloxone used on themselves. Self-report variables around SSP utilization included barriers to access, reasons for re-use of needles/syringes and other drug injection equipment.

Additional variables such as having a primary care provider (PCP), prior infectious complications, history of sexually transmitted infections and viral hepatitis were collected by a combination of self-report and EHR data. *Hepatitis C (HCV) exposure* was defined as positive if the participant self-reported history of HCV, HCV found in EHR, HCV antibody positive with or without HCV RNA. *Hepatitis B (HBV) infection* was defined as self-reported HBV, HBV listed in EHR, positive hepatitis B surface Ag, or positive HBV DNA. *HIV infection* was defined as self-reported HIV infection, positive HIV antigen/antibody or HIV noted in EHR. *Vaccinations* for Hepatitis A, HBV, and Tdap were collected via self-report and EHR. *Rurality* was categorized as either rural (small or isolated rural) or urban (large rural or metropolitan) using the Rural–urban commuting area (RUCA) codes [2]. Other variables were collected through EHR review exclusively. Such variables included insurance status, infectious disease diagnosis, Charlson co-morbidity index [6], prescribed MOUD prior to admission. *MOUD prior to admission* was defined as buprenorphine, buprenorphine/naloxone, naltrexone, or methadone on the pre-admission medication list.

### Statistical analyses

Descriptive analyses were performed to characterize injection knowledge, attitudes and practices. The primary outcome was past 3-month SSP utilization, and the main independent variable was distance to closest SSP. Data were compared between subgroups using t-tests for continuous data; Pearson’s Chi square tests or Fisher’s exact tests were used as appropriate for categorical data. Logistic regression analyses were performed to identify factors associated with SSP utilization. Potential covariates were chosen a priori based on clinical knowledge and literature review. Bivariate unadjusted odds analyses were performed testing the following variables: gender, insurance, employment status, homelessness, overdose history, PCP, insurance, condomless sex, distance, MOUD, trouble accessing SSP, HIV, HBV, HCV, SIP-DU and BIRSI scores**.** SAS Enterprise Guide version 7.1 was used for the analysis.

## Results

### Descriptive analysis

There were 101 study participants enrolled in the study, of whom n = 65 (64%) reported SSP utilization (Table 1). Fifty-six percent (n = 57) of participants had a distance of ≥ 10 miles to the closest SSP. Few participants used engaged in safe practices each time they injected (Table 2). Ninety-four percent of participants reported licking the tip of their needle prior to injecting.

**Table 1:** Demographics and health characteristics among the study population, stratified by syringe service program use

	Overall n = 101	SSP use^a^ n = 65	No SSP use n = 36	*p* value^b^
*Demographics*				
Female^c^	56 (55%)	40 (62%)	16 (44%)	0.10
Mean age (SD)	36 (7)	36 (8)	36 (6)	0.94
Caucasian	95 (94%)	61 (94%)	34 (94%)	1.0
Insurance^d^				
Medicaid	60 (61%)	39 (61%)	21 (60%)	0.13
Medicare	6 (6%)	6 (9%)	0	
Dual Medicare/Medicaid	3 (3%)	3 (5%)	0	
Commercial	5 (5%)	2 (3%)	3 (9%)	
Uninsured	25 (25%)	14 (22%)	11 (31%)	
Primary care provider	68 (67%)	48 (74%)	20 (56%)	0.06
History of incarceration^c^	90 (89%)	61 (94%)	29 (81%)	0.05
Experiencing homelessness	46 (46%)	36 (55%)	10 (28%)	0.01
Small/Isolated rural	18 (18%)	5 (8%)	13 (36%)	< 0.01
> 10 miles from SSP	57 (56%)	28 (43%)	29 (81%)	< 0.01
*Health characteristics*				
Person with HIV	1 (1%)	1 (1.5%)	0 (0)	0.61
Hepatitis B infection	8 (8%)	6 (9%)	2 (6%)	0.71
Hepatitis C exposure	73 (72%)	50 (77%)	23 (64%)	0.16
Condomless sex	76 (75%)	46 (71%)	30 (83%)	0.16
Pregnant	3 (3.0%)	2 (3%)	1 (3%)	1.0
History of infectious complications				
Endocarditis	52 (51%)	36 (55%)	16 (44%)	0.29
Skin/soft tissue infection	31 (31%)	21 (32%)	10 (28%)	0.64
Septic emboli	26 (26%)	19 (29%)	7 (19%)	0.28
Bacteremia/sepsis	18 (56%)	14 (22%)	6 (17%)	0.56
Septic joint	11 (11%)	7 (11%)	4 (11%)	1.0
Epidural abscess	14 (14%)	5 (8%)	9 (25%)	0.03
Osteomyelitis/Diskitis	26 (26%)	13 (20%)	13 (36%)	0.08
History of sexually transmitted infection	27 (27%)	21 (32%)	6 (17%)	0.09
Any mental health condition	91 (90%)	62 (95%)	29 (81%)	0.03
Charlson comorbidity index (1 or more)	25 (24%)	15 (23%)	10 (28%)	0.60
Hepatitis A vaccine completion	58 (58%)	39 (61%)	19 (54%)	0.52
Hepatitis B vaccine completion	53 (53%)	37 (58%)	16 (44%)	0.20
Tdap vaccination	43 (43%)	33 (51%)	10 (28%)	0.03

^a^SSP = syringe service program; ^b^Pearson’s chi-squared; Fisher’s exact test; t-test; ^c^n = 1 female to male transgender participant identified as male; thus, was categorized as male; ^d^missing n = 2

**Table 2:** Substance use characteristics and syringe acquisition/disposal in study participants, stratified by syringe service program use

	Overall n = 101 (%)	SSP use^a^ n = 65 (%)	No SSP use n = 36 (%)	*p* value^b^
*Substance use characteristics*				
Drug use severity (mean SIP-DU^c^ (standard deviation))	33 (12)	33 (12)	32 (13)	0.71
Unhealthy alcohol use (positive AUDIT-C score)^d^	31 (31%)	17 (26%)	14 (39%)	0.20
Skin infection risk (median BIRSI-7 score^e^)	3.8 (1.6)	3.75 (1.5)	3.81 (1.8)	0.88
Injection drugs of choice				0.03
Heroin	39 (40%)	32 (49%)	7 (21%)	
Fentanyl	14 (14%)	7 (11%)	7 (21%)	
Cocaine	11 (11%)	7 (11%)	4 (12%)	
Amphetamine	11 (11%)	9 (14%)	2 (6%)	
Buprenorphine	8 (8%)	3 (5%)	5 (15%)	
Speedball (cocaine + heroin)	9 (9%)	5 (8%)	4 (12%)	
Other	6 (6%)	2 (3%)	4 (12%)	
History of overdose	54 (53%)	42 (65%)	12 (33%)	< 0.01
MOUD^f^ before admission	67 (66%)	46 (71%)	21 (58%)	0.20
Naloxone uptake	48 (48%)	36 (55%)	12 (33%)	0.03
Injected alone 30 days prior to hospitalization	89 (91%)	60(92%)	29 (88%)	0.50
*Syringe acquisition and disposal*				
Used new needle always/most of the time	42 (42%)	34 (52%)	8 (22%)	0.05
Used clean works (cookers, filters) always/most of the time	24 (24%)	20 (31%)	4 (11%)	0.04
Where needles acquired^g^				< 0.01
SSP	39 (39%)	38 (59%)	1 (3%)	
Pharmacy	51 (51%)	23 (35%)	28 (80%)	
Peer exchange	9 (9%)	3 (5%)	6 (17%)	
Interest in mobile harm reduction unit	97 (97%)	65 (100%)	32 (91%)	0.04
Interest in supervised injection facility	93 (93%)	62 (95%)	31 (89%)	0.24

^a^SSP = syringe service program; ^b^Pearson’s chi-squared; Fisher’s exact test; t-test; ^c^SIP-DU = Short-inventory of Problems-Modified for Drug Use; ^d^AUDIT-C = Alcohol Use Disorders Identification Test; ^e^Bacterial Skin Index Risk Score; ^f^Medication for opioid use disorder; n = 14 prescribed methadone, n = 45 buprenorphine or buprenorphine/naloxone, n = 8 self-reported prescription for either methadone, buprenorphine, or buprenorphine/naloxone; no participants self-reported naltrexone; ^g^n = 1 other

Differences in demographics in participants with SSP utilization versus no SSP utilization include homelessness (55% versus 28%, *p* = 0.01), distance ≥ 10 miles (43% versus 81%, *p* < 0.01), and rurality (8% versus 36%, *p* < 0.01). There were no significant differences in SSP utilization by race, gender, sexual preference, or insurance status.

In terms of health differences, participants with a mental health condition were more like to report SSP utilization compared to those who did not use SSPs (95% versus 81%, *p* = 0.03). History of overdose was more common among participants who reported SSP utilization (65% versus 33%, *p* < 0.01). Overall, most participants reported opioids as injection drugs of choice; notably, 30% of participants reported stimulants (amphetamine, cocaine, cocaine plus heroin) as their drugs of choice. There were significant differences in injection drugs of choice among participants with SSP utilization (notably more methamphetamine and heroin use) compared to participants who did not use SSPs (Table 2). Certain infectious complications, such as epidural abscesses, were less common among participants with SSP utilization. Naloxone uptake was more prevalent among participants with SSP utilization (55% versus 33%, *p* = 0.03). Preventive and treatment services such as Tdap, hepatitis A and B vaccination and MOUD treatment were higher among participants who reported SSP utilization. There were no statistically significant differences in other health characteristics or substance use characteristics between the two groups (HCV, HIV, pre-exposure prophylaxis awareness, skin and soft tissue infection risk (BIRSI-7 score), drug use severity (SIP-DU), or unhealthy alcohol use (AUDIT-C) (Table 2).

There were several differences in syringe acquisition and disposal between the two groups. People who reported SSP utilization were more likely to use clean “works” (i.e. cookers and filters; (31% versus 11%, *p* = 0.04) and clean needles (52% versus 22%, *p* = 0.05) all or most of the time in the past 3 months. They were also more likely neutral/interested in mobile syringe service program units or supervised injection facilities. There were no statistically significant differences in syringe disposal in public places (45% versus 37%, *p* = 0.6), re-use of needles (82% versus 78%, *p* = 0.8), or *always* using clean needles (12% versus 6%, *p* = 0.6) or always using clean works (5% versus 6%, *p* = 0.24). Participants who did not use SSPs were more likely to acquire their needle/syringes from pharmacies or peer exchange (Table 2).

Of those n = 65 participants who used an SSP, 33% used an SSP frequently (few times per week), whereas others used it sometimes (21%) or hardly ever (28%). Many reported using one because its free services (57%) or because of privacy (31%). Fifty-four percent of participants reported trouble accessing an SSP, largely due to distance (28%), lack of car (19%), or stigma (14%).

### Multivariable analysis

Gender, homelessness, history of overdose, having primary care physician and distance to SSP were included in the final multivariable regression model based on statistical significance of *p* < 0.05 in the bivariate, unadjusted odds analyses. Participants who lived ≤ 10 miles from an SSP were more likely to use an SSP (OR 5.4, 95% CI 1.9–15.7), controlling for female gender, homelessness, history of overdose, and having a PCP (Table 3). Notably, participants with history of overdose (OR 3.0, 95% CI 1.1–8.1) and with a PCP (3.1, 95% CI 1.1–8.7) were also more likely to use an SSP.

**Table 3:** Unadjusted and adjusted analyses of predictors of syringe service program use

Variable	Unadjusted OR (95% CI)	Adjusted OR (95% CI)
Female (ref^a^ = male)	2.0 (0.9–4.6)	1.4 (0.5–3.6)
Homelessness (ref = housed)	3.2 (1.3–7.8)	2.5 (0.9–7.0)
History of overdose (ref = none)^b^	3.0 (1.5–8.6)	3.0 (1.1–8.1)
Primary care physician (ref = none)^b^	2.3 (1.0–5.3)	3.1 (1.1–8.7)
Driving distance ≤ 10 miles (ref = > 10 miles)^b^	5.5 (2.1–14.3)	5.4 (1.9–15.7)

^a^ref = reference group; ^b^denotes statistical significance; *p* < 0.05 considered statistically significant

## Discussion

Prior studies have documented the health benefits of access to SSPs, particularly when combined with access to MOUD treatment [7, 18]. Given the unsafe injection practices in this study population and geographic distribution of PWID in rural Maine, expansion of SSPs, including mobile units, could improve SSP utilization [28] and potentially prevent IDU-associated infections [8]. Thus far, behavioral interventions that include harm reduction counseling in the hospital have not yet been shown to reduce future hospitalizations [27], leading to an emphasis on community strategies for harm reduction, such as access to SSPs. At the time of this study, there were only 6 SSPs in Maine, though in the setting of increasing overdoses and costly IDU-associated infections, additional SSPs opened upon study completion. It is noteworthy that living in rural settings can lead to barriers to access to SSPs due to both distance and to lack of public transportation. Our study results are consistent with prior research where geographic disparities in SSP access were noted, particularly among young people with acute HCV [5]. Modeling studies have shown that scaling up prevention (SSPs) and treatment efforts (MOUD) could decrease the burden of IDU-associated infections like HCV [9, 18]. In our study, 66% of the participants were being treated with MOUD, yet still developed IDU-associated infections. Given rising stimulant use [11] and lack FDA-approved pharmacotherapy for stimulant use disorder, our findings highlight the importance of preventive services like SSPs in addition to expanding treatment and recovery services. While a multipronged approach including Medicaid expansion, increasing treatment capacity, housing security, and access to pre-exposure prophylaxis for HIV, is certainly needed for infection prevention [26], increased access to SSPs is especially important given the disproportionate burden of IDU-associated infections in rural areas.

While SSPs help reduce infection transmission, they also play a large role in naloxone distribution and overdose reversals [23]. Our results also showed that participants with a history of overdose were more likely to use SSPs. Notably, 90% of our study population reported injecting alone in the 30 days prior to hospitalization, and 72% of the participants supported supervised injection facilities, which are safe places where people can inject pre-owned drugs. While increasing accessibility to SSPs is needed, particularly to people who may not perceive overdose risk, consideration of supervised injection facilities is also important given the overdose and infection risks in our study population. Sanctioned supervised injection facilities have not yet been adopted in the United States at the time of this study, but have been shown to reduce adverse outcomes, such as overdoses, elsewhere [13].

This study had some limitations. Nearly 20% of the study population came from small/isolated rural areas, and there was only one participant with HIV; our results may not be generalizable to more urban regions or regions with a higher prevalence of HIV. While reflective of our state’s demographics, a majority of our study population was white. Black, Indigenous, people of color may experience additional barriers to SSP utilization that were not captured by this study. Selection bias also likely explains our findings that participants with history of overdose were more likely to use SSPs. Also, the high rate of unsafe injection practices in our convenience sample is likely due, in part, to a selection bias implicit in our study population, whose practices around safe injection techniques may not represent non-hospitalized people who use SSPs.

## Conclusions

In our study of people hospitalized with IDU-associated infections in a rural state, unsafe injection practices were common. Especially given increasing stimulant use, these results also highlight the need to improve SSP access even among individuals prescribed medication for opioid use disorder. Living ≤ 10 miles from an SSP was associated with increased SSP utilization. Expansion of SSPs into rural areas, including mobile units, could increase SSP utilization and potentially reduce morbidity and mortality.

## Data Availability

De-identified data can be made available upon request to corresponding author.
